# Antioxidant, Antimicrobial, and Bioactive Potential of Two New Haloarchaeal Strains Isolated from Odiel Salterns (Southwest Spain)

**DOI:** 10.3390/biology9090298

**Published:** 2020-09-18

**Authors:** Patricia Gómez-Villegas, Javier Vigara, Marta Vila, João Varela, Luísa Barreira, Rosa Léon

**Affiliations:** 1 Laboratory of Biochemistry, Department of Chemistry, University of Huelva, Avda. de las Fuerzas Armadas s/n, 21071 Huelva, Spain; patgomvil@gmail.com (P.G.-V.); vigara@uhu.es (J.V.); marta.vila@dpcm.uhu.es (M.V.); 2 Centre of Marine Sciences, University of Algarve, Campus of Gambelas, 8005-139 Faro, Portugal; jvarela@ualg.pt (J.V.); lbarreir@ualg.pt (L.B.)

**Keywords:** antioxidant, anti-inflammatory, antimicrobial, bioactive substances, haloarchaea

## Abstract

**Simple Summary:**

Halophilic archaea are microorganisms that inhabit in extreme environments for life, under salt saturation, high temperature and elevated UV radiation. The interest in these microorganisms lies on the properties of their molecules, that present high salt and temperature tolerance, as well as, antioxidant power, being an excellent source of compounds for several biotechnological applications. However, the bioactive properties from haloarcahaea remain scarcely studied compared to other groups as plants or algae, usually reported as good health promoters. In this work we describe the isolation and the molecular identification of two new haloarchaeal strains from Odiel salterns (SW Spain), and the antioxidant, antimicrobial and bioactive potential of their extracts. The results revealed that the extracts obtained with acetone presented the highest activities in the antioxidant, antimicrobial and anti-inflammatory assays, becoming a promising source of metabolites with applied interest in pharmacy, cosmetics and food industry.

**Abstract:**

The need to survive in extreme environments has furnished haloarchaea with a series of components specially adapted to work in such conditions. The possible application of these molecules in the pharmaceutical and industrial fields has received increasing attention; however, many potential bioactivities of haloarchaea are still poorly explored. In this paper, we describe the isolation and identification of two new haloarchaeal strains from the saltern ponds located in the marshlands of the Odiel River, in the southwest of Spain, as well as the in vitro assessment of their antioxidant, antimicrobial, and bioactive properties. The acetone extract obtained from the new isolated *Haloarcula* strain exhibited the highest antioxidant activity, while the acetone extracts from both isolated strains demonstrated a strong antimicrobial activity, especially against other halophilic microorganisms. Moreover, these extracts showed a remarkable ability to inhibit the enzyme cyclooxygenase-2 and to activate the melanogenic enzyme tyrosinase, indicating their potential against chronic inflammation and skin pigmentation disorders. Finally, the aqueous protein-rich extracts obtained from both haloarchaea exhibited an important inhibitory effect on the activity of the acetylcholinesterase enzyme, involved in the hydrolysis of cholinergic neurotransmitters and related to several neurological diseases.

## 1. Introduction

Halophilic archaea or haloarchaea are a group of extremophilic microorganisms inhabiting hypersaline environments, such as salt lakes and salterns. They thrive in harsh conditions for life, including low water availability, high salt concentration, and elevated solar irradiance, which implies high temperature and ultra-violet (UV) radiation [1,2]. The biotechnological interest in the haloarchaeal group has increased due to their ability to produce a wide variety of compounds such as exopolysaccharides, carotenoids, and proteins adapted to work in such extreme conditions. The tolerance of these enzymes to elevated saline concentrations and temperatures, the high antioxidant power and therapeutic potential of haloarchaeal carotenoids or the good jellifying properties and thermal stability of the exopolysacharides secreted by many archaea make these metabolites highly appreciated for numerous biotechnological applications, including biomedical, pharmaceutical, cosmetic, environmental or industrial purposes [3,4]. Furthermore, considering that only a small part of the existing archaeal species has been discovered and studied, it is expected that many other metabolites with unexplored bioactivities can be obtained from this extraordinary group of microorganisms, as pointed in recent reviews [5].

Haloarchaea have shown to be particularly suitable for the production of extremozymes, which can tolerate high temperatures and saturating salt concentrations, conditions required in food, detergent and textile sectors [6,7]. In addition, a common feature within the haloarchaeal group is their ability to secrete halocins, which are peptides or proteins able to inhibit the growth of susceptible microorganisms living in the same habitat [8,9]. These molecules are also resistant to extreme conditions and could be a source of new antimicrobial compounds. Moreover, most haloarchaea can produce and accumulate carotenoids, which are responsible for their red color. Carotenoids are antioxidant compounds with an important role as human health enhancers, and it is widely known that their regular consumption helps to prevent many degenerative diseases, such as neurodegeneration, cancer, cardiovascular diseases, macular degeneration and cataracts [10,11].

Carotenoids from haloarchaea have shown anticancer and antihemolytic activities [12], and the ability to increase sperm cells viability after cryopreservation [13]. Furthermore, many natural carotenoids are used as food preservatives and pharmaceutical and cosmetic compounds due to their antioxidant and pro-vitamin properties [14]. Bacterioruberin (BR) and its derivatives, monoanhydrobacterioruberin (MABR) and bisanhydrobacterioruberin (BABR), are the main carotenoids synthesized by halophilic archaea [15,16,17]. These carotenoids have 50 carbon atoms (C_50_) and possess a longer system of conjugated double bonds than the C_40_ carotenoids usually found in other organisms such as plants, microalgae, fungi and bacteria [14]. Some C_40_ carotenoids, such as phytoene, lycopene and β-carotene have also been found in haloarchaea, but at such low quantities that they have been proposed as metabolic intermediates for the biosynthesis of C_50_ carotenoids [18,19]. Even though carotenoids play a unique important role in cell protection against oxidative damage, bacterioruberin appears also to be a stabilizer of the cell membrane under osmotic stress [20]. Because of the structure of bacterioruberin, it can be inferred that its antioxidant potential might be even higher than that of β-carotene, due to the fact that it has 13 conjugated double bonds and four hydroxyl groups, compared to the 9 conjugated double bonds and none hydroxyl group of β-carotene [16]. For those reasons, the antioxidant power of haloarchaeal extracts and their potential as alternative to current food preservatives and as drug leads should be more deeply studied.

Pigments from different kinds of marine microorganisms have shown effective antibacterial properties. Examples of this are the red pigment prodigiosin, isolated from the sponge-associated bacterium *Serratia marcescens* [21]; or the chlorophyll a derivatives obtained from the microalga *Isochrysis galbana* [22]. Recently, the antimicrobial activity of pigments from some halophilic bacteria has been tested against bacteria and fungi [23]; however, so far, the studies about the antimicrobial activity of haloarchaeal pigments or extracts thereof are scarce [19,24].

The main aim of this work is to explore the bioactive properties of different extracts obtained from two new strains of haloarchaea isolated from the Odiel solar saltern (SW Spain), focusing on their antioxidant and antimicrobial activities; and their capability to modulate the activity of disease-related enzymes involved in melanin biosynthesis, neurological degeneration, carbohydrate metabolism, and inflammatory response, through in vitro assays.

## 2. Materials and Methods

### 2.1. Sample Collection

Water samples were collected from a crystallizer pond located in the natural reserve of the Odiel Marshlands, in the southwest of Spain (Latitude: 37.2395, longitude: −6.95287). The ionic composition and the physicochemical parameters of the water brine were determined by the standard methods, as previously reported [20].

### 2.2. Archaea Isolation

The biomass from 2 L of the collected water samples was harvested by centrifugation at 19,800× *g*, resuspended in 20 mL of archaeal medium (ATCC 1176 medium) and used for the isolation of archaeal colonies on agar plates. Typically, 1 mL of serial dilution corresponding to 1:10, 1:100 and 1:1000 were spread on agar medium containing 20% of salt. After incubation at 37 °C for 15 days, red and pink colonies were visible. Selected colonies were purified by at least three streaking rounds on fresh agar plates. The isolates were preserved in 20% glycerol (w/v) at −80 °C for further use.

### 2.3. Preliminary Selection of Archaeal Strains with Antimicrobial Activity

Halocin activity was determined by observing growth inhibition of the archaea *Haloferax lucetense* (CECT 5871), which was purchased from CECT (Spanish Collection of Culture Type, Valencia, Spain) and whose susceptibility to the halocin activity of the species inhabiting Odiel salterns was previously probed [25]. *H. lucetense* was grown on the medium specified by the CECT (MHE 25 Medium; CECT 188), and 1 mL of the culture was completely spread across the surface of a Petri dish. When the plate was totally dried, a 20 µL drop of the culture of each previously isolated colony was spotlessly placed onto the Petri dish. The inhibition of *H. lucetense* growth was measured by the formation of a clearance zone around each drop added.

### 2.4. Identification of the Selected Microorganisms

Genomic DNA of each strain was extracted using the GeneJET Genomic Purification kit (Thermo Fisher Scientific, Waltham, MA, USA), following the manufacturer’s instructions. Quantification of the genomic DNA obtained, and the assessment of its purity was done on a Nanodrop Spectrophotometer ND-1000 (Thermo Fisher Scientific). The full length of the 16S rRNA encoding gene was amplified with the archaeal specific primers 21F (5′-TTCCGGTTGATCCTGCCGGA-3′) and 1492R (5′-GGTTACCTTGTTACGACTT-3′). Polymerase chain reactions (PCR) were performed in a total volume of 25 µL containing: 1 µL of genomic DNA, 10 pM of each primer, 0.2 mM dNTPs, 0.2 U REDTaq® DNA polymerase from Sigma Aldrich (St. Louis, Missouri, USA) 2.5 µL of specific 10X buffer and 1.5 µL of 2.5 mM MgCl_2_ buffer using an Eppendorf thermo-cycler. The thermal profile corresponded to 0.5 min at 96 °C, 0.5 min at 55 °C and 1 min at 72 °C for 30 cycles, followed by 10 min of final primer extension. The PCR products were analyzed by electrophoresis on a 1 % agarose gel to check their quality and sent to Stabvida (Lisbon, Portugal) for Sanger sequencing. The 1.4 kb 16S rRNA gene sequences obtained were compared to those available at the GenBank and the European Molecular Biology Laboratory (EMBL) databases using advanced Basic Local Alignment Search Tool (BLAST), Megablast and BLASTn, searches at the National Center for Biotechnology Information (NCBI) [26].

### 2.5. Archaeal Culture Conditions

The medium used for haloarchaeal growth contained (per liter): 10 g glucose, 156 g NaCl, 13 g MgCl_2_·6H_2_O, 20 g MgSO_4_·7H_2_O, 1 g CaCl_2_·6H_2_O, 4 g KCl, 0.2 g NaHCO_3_, 0.5 g NaBr and 5 g yeast extract. The pH was adjusted to 7 before autoclaving. The cultures were incubated in Erlenmeyer flasks at 37 °C at 100 rpm for one week. 

### 2.6. Preparation of Archaeal Extracts

When cultures reached the stationary phase of growth, 1 L of each culture was collected by centrifugation at 19,800× *g* for 30 min at 4 °C. The obtained cellular pellets were subjected to five freezing-unfreezing cycles, by successive 1 min immersions in liquid nitrogen/hot water (60 °C). The obtained cell lysates were treated overnight with 100 mL of cold acetone (−20 °C) in the dark. Then, acetone extracts were centrifuged at 4 °C and 19,800× *g* for 30 min. The resulting pellet, containing the proteins precipitated with cold acetone and the acetone supernatant, containing carotenoids and other lipophilic compounds, were dried in a rotary vacuum evaporator and freeze-dried for further study. The total content of carotenoids in acetone extracts was determined by measuring the absorbance of the sample at 494 nm in a cuvette with a 1 cm path length and using the specific absorption coefficient E_1_^1%^ = 2500 (100 mL g^−1^ cm^−1^) according to Hiyana et al. [27]. Similarly, the concentration of proteins in the acetone precipitated extracts, hereinafter called aqueous extract, was determined by Bradford’s method [28].

Potential bioactive compounds excreted into the culture medium by the selected archaea were recovered from 500 mL of each culture medium by successive liquid/liquid extractions with 50 mL of four organic solvents in this order: hexane, dichloromethane, ethyl acetate, and chloroform. The extraction with each solvent was repeated three times. All the fractions were evaporated to dryness in a rotary evaporator under reduced pressure at 50 °C, freeze-dried, and stored until further use.

### 2.7. Antioxidant Activity Assays

Different assays were conducted to test the antioxidant activity of archaea cell extracts and all the extracellular extracts obtained from the culture medium, as previously described. Each freeze-dried extract was homogenized and dissolved in DMSO (at 10 mg mL^−1^) except for the acetone precipitate, which was resuspended in phosphate-buffered saline (PBS) buffer. All samples were tested at a concentration of 1 mg mL^−1^. Butylated hydroxytoluene (BHT, 1 mg mL^−1^) was used as positive control in the 1,1-Diphenyl-2-picrylhydrazyl (DPPH), 2,2′-azino-bis(3-ethylbenzothiazoline-6-sulfonic acid (ABTS) and nitric oxide (NO) radical scavenging assays, and in the ferrocyanide reduction potential (FRP), while the metal chelator EDTA (1 mg mL^−1^) was employed as positive control in the copper (CCA) and iron (ICA) chelating assays. The solvent employed to dissolve the extracts (DMSO or PBS) was used as negative control. All the measurements were carried out at least six times on 96-well plates. The radical scavenging activity was expressed as percent of inhibition relative to the negative control, following the formula:

$$\%\;Scavenging\;activity=100-\;\frac{Abs\;sample\times 100}{Abs\;negative\;control}$$

When the activity of a sample was higher than 65%, the concentration of the extract providing 50% reduction of the radical scavenging activity (IC_50_ or EC_50_) was calculated by sigmoidal fitting of the data, using the GraphPad Prism 6 program. These values were calculated from a graph built by plotting the log of the radical scavenging activity (%) against the normalized sample concentration, according to the concentration of proteins or carotenoids in the extracts.

#### 2.7.1. DPPH Assay

1,1-Diphenyl-2-picrylhydrazyl radical scavenging assay was performed by the method of Sachindra et al. [29] with some modifications. A solution of DPPH 0.12 mM was prepared in methanol; 200 μL of this DPPH solution were mixed with 22 μL of each extract solution. The absorbance decrease at 517 nm was recorded in a microplate reader, after incubation at 25 °C for 30 min in the dark. A color control prepared with 200 μL of methanol and 22 μL of each extract sample was included in all the assays.

#### 2.7.2. ABTS Assay

Free radical scavenging activity of the extracts was determined by the ABTS^+^ (2,2′-azino-bis(3-ethylbenzothiazoline-6-sulfonic acid)) radical cation decolorization assay [30]. ABTS^+^ cation radical was generated by the reaction between ABTS (7 mM in water) and potassium persulfate (2.5 mM), in the dark at room temperature for 12–16 h. The ABTS^+^ solution was then diluted with methanol to obtain an absorbance of 0.700 at 734 nm. After the addition of 10 μL of each extract to 190 µL of the diluted ABTS^+^ solution, the samples were incubated for 6 min in the dark and the absorbance at 734 nm was measured after the incubation.

#### 2.7.3. Nitric Oxide (NO) Assay

The capacity to scavenge the free radical NO was evaluated according to Baliga et al. [31]. Briefly, 50 µL of extracts were mixed with 50 µL of sodium nitroprusside (10 mM in PBS) and incubated for 90 min at room temperature before adding 50 µL of Griess reagent. After that, the absorbance was measured at 546 nm.

#### 2.7.4. Ferrocyanide Reducing Power (FRP) Assay

The reducing power assay was performed according to Tundis et al. [32]. The assay solution was prepared by mixing 50 µL of sample solution, 50 µL of distilled water and 50 µL of potassium ferrocyanide (1%). After 20 min of incubation, 50 µL of TCA (10%) and 10 µL of FeCl_3_ (0.1%) were added and the mix was incubated 10 min before measuring the absorbance at 700 nm.

#### 2.7.5. Metal Chelating Activity on Iron and Copper Ions

Both assays were conducted according to Megías et al. [33]. Iron chelating activity (ICA) was determined by measuring the formation of the Fe^2+^-ferrozine, by measuring the absorbance at 562 nm. Copper chelating activity (CCA) was determined using pyrocatechol violet (PV), detecting the color change at 632 nm.

### 2.8. Inhibition of Carbohydrate-Hydrolyzing Enzymes

The ability of the obtained extracts to inhibit the carbohydrate-hydrolyzing enzymes *α*-amylase and *α*-glucosidase was tested by incubating dilutions of the haloarchaeal extracts (1 mg mL^−1^), prepared in sodium phosphate buffer 0.1 M pH 7, with the corresponding enzymes. Acarbose (1 mg mL^−1^) was used as positive control and the buffer was employed as negative control. A blank, made with the buffer without the corresponding assayed enzyme, was also included. All the assays were conducted in 96 well plates and repeated at least six times. The inhibitory activity was calculated according to the formula:

$$\%\;Inhibitory\;activity=100\;\times \;\frac{Abs\;sample-Abs\;blank}{Abs\;negative\;control}$$

#### 2.8.1. α-Amylase Inhibition Test

The α-amylase inhibition assay was performed as previously described by Iauk et al. [34]. Briefly, 40 µL of sample were mixed with 40 µL of amylase solution (100 U mL^−1^ in buffer) and 40 µL of starch solution (0.1% in buffer sodium phosphate buffer 0.1M pH 7) and incubated 10 min at 37 °C. After that, 20 µL of HCl (1 M) and 100 µL of iodide solution (5 mM I_2_ + 5 mM KI, in dH_2_O) were added and the absorbance at 580 nm was read.

#### 2.8.2. α-Glucosidase Inhibition Test

The α-glucosidase inhibition assay was performed according to Iauk et al. [34]. Concisely, 50 µL of sample were added to 100 µL of enzyme solution (1.0 U mL^−1^) and incubated 10 min at room temperature. Then, 50 µL of 5mM p-nitrophenyl-a-D glucopyranoside was added and, after another incubation of 5 min at 25 °C, the absorbance was read at 405 nm.

### 2.9. Acetylcholinesterase (AChE) Activity

The AChE activity was measured by the Ellman method as described by Orhan et al. [35]. Typically, 20 µL of each sample (10 mg mL^−1^ in DMSO) were mixed with 140 µL of 0.1 mM sodium phosphate buffer (pH 8.0) and 20 µL of an AChE (EC.3.1.1.7) solution (0.28 U mL^−1^) from electric eel, and incubated at room temperature for 15 min. The reaction was started by adding 10 µL of ATChI (acetylthiocholine iodide, 4 mg mL^−1^) together with 20 µL of DTNB (5,5-dithiobis-2-nitrobenzoic acid, 1.2 mg mL^−1^). The hydrolysis of ATChI was monitored at 412 nm, by the formation of the yellow 5-thio-2-nitrobenzoate anion as a result of the reaction between DTNB and the thiocholine produced from the reaction catalyzed by the enzyme. Results were expressed as percentages of activity relative to the negative control, as indicated above. Galantamine (1 mg mL^−1^) was used as positive inhibitory control, while the sample solvent was set as the negative control. All the reactions were carried out in 96-well microplates and repeated at least six times.

### 2.10. Tyrosinase (TYRO) Activity

The effect of the different extracts on the tyrosinase activity was determined, by the method reported by Nerya et al. [36] with some modifications. Essentially, 70 µL of samples (10 mg mL^−1^) dissolved in phosphate buffer 25 mM pH 6.5 were mixed with 30 µL of TYRO (333 U mL^-1^ in phosphate buffer, pH 6.5) and incubated for 5 min. After that, 110 µL of substrate (L-tyrosine, 2 mM in water) were added and further incubated for 30 min at room temperature, before reading the optical densities at 492 nm. The assays were done with six replicates for each sample in 96 well plates and arbutin (1 mg mL^−1^) was used as positive control. Results were expressed as percentage of activity relative to a negative control as previously detailed, containing the buffer in place of the sample.

### 2.11. Cyclooxygenase 2 (COX-2) Activity

The anti-inflammatory activity of the extracts was studied by testing the inhibition of the enzyme cyclooxygenase 2 (COX-2), using the fluorometric COX-2 Inhibitor Screening Kit, Biovision K547-100 (Life Science, Milpitas, CA, USA). This assay is based on the fluorometric detection of Prostaglandin G2, the intermediate product generated by the COX enzyme from arachidonic acid. All the extracts were tested at a concentration of 1 mg mL^−1^ and solved in PBS, except the acetone ones, which were prepared in acetone to obtain a better solubility. All the assays were conducted in triplicate, following the manufacturer’s indications. The reactions were started by adding 10 µL of the provided arachidonic acid, using 10 µL of the samples in a final volume of 100 µL of reaction mixture. Celecoxib (2 mM) was employed as positive control and the corresponding solvents, PBS and acetone, were used as negative control of COX-2 inhibition. The fluorometric detection (Ex/Em = 535/587 nm) was recorded in a fluorimeter FLUOstar Omega, BMG LABTECH, and the percentage of inhibition was calculated from the slope for all samples, including Enzyme Control (EC) as follows:

$$\%\;Relative\;Inhibition=\frac{Slope\;of\;EC-Slope\;of\;Sample}{Slope\;of\;EC}\times 100$$

### 2.12. Antimicrobial Activity

#### 2.12.1. Strains and Media

The antimicrobial activity of the extracts obtained from the two selected haloarchaea strains was tested against different kinds of microorganisms including bacteria, microalgae, yeast, and archaea. The bacteria included in the assays were fish pathogens, kindly provided by the Andalusian Institute of Agriculture research: *Streptococcus parauberis* DSM 6631T, *Lactococcus garvieae* CECT 4531T, *Tenacibaculum maritimum* CECT 4276, *Tenacibaculum soleae* CECT 7292T, *Pseudomonas anguilliseptica* CECT 899T, *Pseudomonas moraviensis* DSM 16007T, *Pseudomonas plecoglossicida* DSM 15088T, *Edwardsiella tarda* CECT 849T, *Edwardsiella tarda* CECT 849T, *Vibrio anguillarum* CECT 522T, *Vibrio harveyi* CECT 525T, *Vibrio tapetis* CECT 4600T, *Aeromonas salmonicida salmonicida* CECT 894T, *Pseudomonas baetica* a390T, *Mycobacterium marinum* CECT 7091T, *Yersinia ruckeri* CECT 4319T, *Photobacterium damsela damselae* CECT 626T; common human pathogenic bacteria: *Micrococcus luteus* CECT 245, *Bacillus cereus* CECT 40 and *Staphylococcus aureus*, provided by the Microbiology and Parasitology Department of the University of Seville and the Gram negative *Escherichia coli* DH5α, related to human pathogenic strains. Among the microalgae, two freshwater species, *Chlorella sorokiniana* and *Chlamydomonas reinhardtii* 21gr, and two hypersaline species, *Dunaliella salina* and *Dunaliella bardawil* were tested. Antifungal activity was assayed against three different yeasts; *Saccharomyces cerevisiae* F13A, *Rhodotorula* sp., and *Rhodospirillum toruloides* 1854, supplied by the University of Algarve. Finally, the inhibitory activity of the obtained extracts against different haloarchaeal genera was studied in: *Haloferax* CECT 5871, *Halogeometricum*, *Halogiper*, *Halorubrum, Haloterrigena*, and *Natrinema*, generously given by the University of Mentouri’s Bothers in Constantine, Algeria. All the tested species are listed in Table 1, in the Results section.

Human pathogenic bacteria were cultured in LB (Luria–Bertani) medium, while fish pathogens were cultured in TSB (Tryptic Soy Broth) medium. Fresh water microalgae were cultured in TAP (Tris-Acetate-Phosphate) medium, whereas halophilic microalgae were cultured in modified Johnson’s medium [37,38]. Finally, YPD (Yeast extract-Peptone-Dextrose) and MHE 25 (CECT 188) media were used for yeast and archaea growth, respectively.

#### 2.12.2. Agar Diffusion Method

The indicator strains were grown in liquid media until they reached the late exponential phase of growth. Then, 1 mL inoculums of approximately 10^7^ UFC mL^−1^ were spread across agar plates with their appropriate growth media. Once cell suspensions were completely dried, a 10 µL drop of each extract (10 mg DW mL^−1^) was added to the plate. The extracts obtained from the culture media were resuspended in water or DMSO/water (1:4), while the precipitate and the supernatant obtained by extraction with acetone were diluted in PBS and acetone/water (1:4), respectively. The absence of toxicity of all the solvents was confirmed before the assays. The inhibition of growth was checked after the corresponding incubation period; 24 h at 37 °C for human pathogenic bacteria; 24 h at 25 °C for fish pathogenic bacteria; 48 h at 25 °C and light (100 µE m^−2^ s^−1^ of intensity) for fresh water microalgae; 7 days at 25 °C and light (100 µE m^−2^ s^−1^ of intensity) for hypersaline water microalgae; 24 h at 30 °C for yeasts; and 7 days at 37 °C for haloarchaea. The antimicrobial activity was determined measuring the size of the inhibition zone around the spotted drops. All the experiments were carried out in triplicate.

#### 2.12.3. MIC Determination

Several dilutions of the active extracts were assayed to determine their Minimal Inhibitory Concentration (MIC) for the most susceptible microorganisms of each tested group using the agar diffusion method, as previously described, to detect the lowest extract concentration able to inhibit the growth of the selected microorganisms.

### 2.13. Statistical Analysis

The results were expressed as the mean ± SD of at least triplicate experiments. The data were submitted to one-way variance analysis (ANOVA) and the differences between means were evaluated by a Duncan’s Multiple Range Test. The data were analyzed using the statistical and data analysis solution for Microsoft Excel (XLSTAT 2020, New York, NY, USA). Differences were considered significant at *p* < 0.05.

## 3. Results and Discussion

### 3.1. Isolation, Selection, and Identification of Haloarchaeal Strains

Two new haloarchaeal strains were isolated from environmental water samples, collected at the end of the summer from a salt evaporation pond located in the natural reserve of Odiel Marshlands, where rivers Tinto and Odiel flow into the Atlantic Ocean in the city of Huelva (SW Spain). The total salinity of the water collected was 33.23 g L^−1^, being the composition of the brine of 1.40 g CaSO_4_, 23.06 g MgSO_4_, 34.08 g MgCl_2_, 265.38 g NaCl, 7.51 g KCl and 0.84 g NaBr per liter.

These strains were chosen among a series of haloarchaeal colonies initially isolated from the water samples by serial dilution and successive streaking rounds on agar plates. Those which showed the most vigorous growth and exhibited the highest halocin activity, preliminary tested against *Haloferax lucentense*, were selected to further investigate their antimicrobial potential against a wide number of pathogenic bacteria and other bioactivities, such antioxidant and anti-inflammatory activities as well as their neuroprotective, melanogenic, or antidiabetic potential properties.

**Figure 1:**
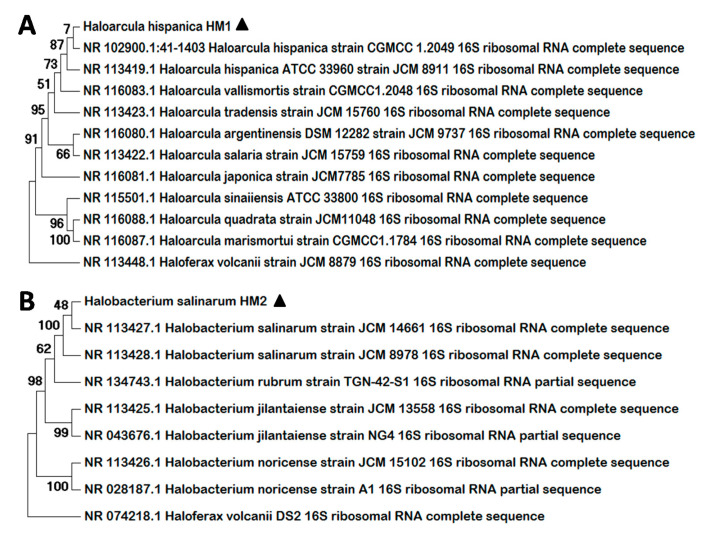
Molecular Phylogenetic Analysis by Maximum Likelihood Method. The trees represent a comparison among the 16S rRNA sequences from the new strains isolated, *H. hispanica* HM1 (**A**) and *H. salinarum* HM2 (**B**), and a series of reference archaeal sequences. Multiple alignments were generated by MUSCLE (MUltiple Sequence Comparison by Log-Expectation) and the trees were constructed with MEGA 7. The numbers at nodes indicate the bootstrap values calculated for 1000 replicates. The name and the NCBI access number are indicated for all the reference sequences. Black triangles indicate the new strains identified.

Amplification of the complete 16S rRNA sequence encoding gene from the new haloarchaeal strains was performed using the primers 21F y 1492R and procedures described in the materials and method section. Comparison of the obtained sequences (Supplementary Materials) with the NCBI database using the BLASTn tool revealed that the two new strains isolated belong to the genus *Haloarcula* and *Halobacterium*, respectively. The first isolate shared a 98.68% of sequence identity with *Haloarcula hispanica,* consequently it was designated as *Haloarcula hispanica* HM1. The 16S rRNA gene of the second haloarchaea isolated, on the other hand, showed high percentage of identity with the corresponding sequence of *Halobacterium salinarum* (97.53%). In addition, a Molecular Phylogenetic Analysis was conducted using the Molecular Evolutionary Genetics Analysis (MEGA) program version 7, including the sequences obtained and the complete sequences of a series of 16S rRNA coding genes available in the NCBI database for several members of the corresponding genus (Figure 1). In both cases, *Haloferax volcanii* was used as outgroup and the bootstrap was set at 1000 replicates. The 16S rRNA encoding sequence of the first isolate forms a cluster with the corresponding genes of *H. hispanica* and is closely related with the sequence of other *Haloarcula* species (Figure 1A). Otherwise, the 16S rRNA sequence of the second haloarchaea isolated clusters together with *H. salinarum* sequences and is highly related with the sequence of several species of the genus *Halobacterium* (Figure 1B).

*Haloarcula* [39,40] and *Halobacterium* [41,42] genera belong to the *Halobacteriaceae* family within the *Halobacteriales* order. Currently, ten recognized species are included in the *Haloarcula* genus: *H. amylolytica* [43], *H. argentinensis* [44], *H. vallismortis* [45], *H. marismortui* [46], *H. hispanica* [47], *H. japonica* [48], *H. quadrata* [49], *H. sinaiiensis* [50], *H. Salaria* and *H. Tradensis* [51]; while to date, five species comprise the *Halobacterium* genus: *H. Jilantaiense* [52], *H. litoreum* [53], *H. noricense* [54], *H. rubrum* [55] and *H. salinarum* [56]. Several strains of these species have been described due to the divergence of their 16S RNA coding gene sequences, as is the case of the two strains proposed here, which have been designated as *Haloarcula hispanica* HM1 and *Halobacterium salinarum* HM2.

### 3.2. Bioactive Properties of the Extracts from H. Hispanica HM1 and H. Salinarum HM2

A total of 12 extracts, six from each of the new isolated haloarchaeal strains, were obtained from cultures of *H. hispanica* HM1 and *H. salinarum* HM2 harvested at the end of the stationary phase of growth. Hexane, dichloromethane, ethyl acetate, and chloroform extracellular extracts were recovered from the culture media, while acetone and aqueous extracts were obtained from the archaea biomass. The potential bioactive properties of all these extracts was tested as detailed below.

#### 3.2.1. Antioxidant Capacity

**Figure 2:**
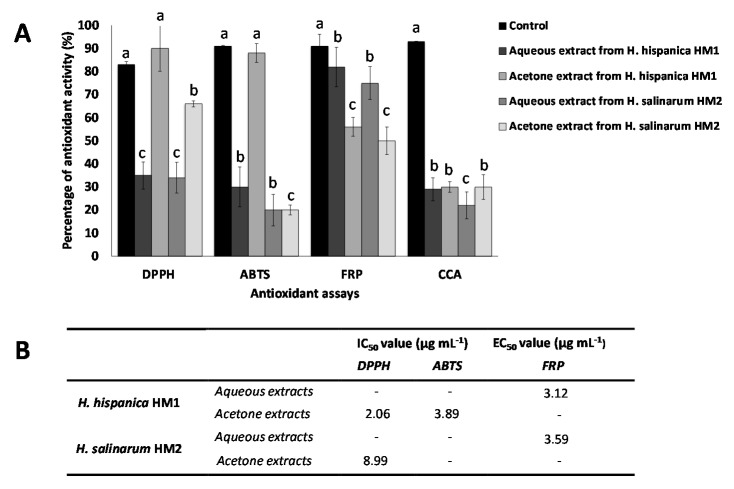
Antioxidant activity of aqueous and acetone extracts (1 mg mL^−1^) obtained from the haloarchaeal strains *H. hispanica* HM1 and *H. salinarum* HM2, determined by DPPH (1,1-Diphenyl-2-picrylhydrazyl) and ABTS (2,2′-azino-bis(3-ethylbenzothiazoline-6-sulfonic acid)) radical scavenging assays, or FRP (Ferrous ion reduction potential) and CCA (Copper chelating activity). For each assay, the data were submitted to one-way variance analysis (ANOVA). Bars are followed by different superscript letters (a, b or c), which denote groups with significant differences according to the Duncan’s Multiple Range Test (*p* < 0.05) (**A**). The half maximal inhibitory or effective concentration (IC_50_ or EC_50_) were calculated when the activity was higher than 65% (**B**). These values were normalized to the content of proteins and carotenoids in the extracts, being the protein concentration of the aqueous extract 47 and 30 µg per mg DW and the carotenoid content of 10 and 12 µg per mg DW, respectively, for *H. hispanica* HM1 and *H. salinarum* HM2.

The antioxidant potential of all the extracts was tested in radical scavenging assays, based on DPPH, ABTS, or NO, by the ferrocyanide reduction potential (FRP), and ICA and CCA metal chelation assays. The half maximal inhibitory concentration (IC_50_) or the half maximal effective concentration (EC_50_) was calculated using the prism program version 6 for the extracts that exhibited antioxidant activities higher than 65% (Figure 2).

None of the extracellular extracts obtained from the culture media was found to have antioxidant activity, while cellular extracts showed a considerable antioxidant activity, being able to scavenge DPPH, and ABTS radicals, reduce ferrocyanide and chelate copper, but not scavenge NO radicals or chelate iron. The aqueous extracts obtained from *H. hispanica* HM1 and *H. salinarum* HM2 biomass showed a moderate antioxidant capacity on DPPH, ABTS, and CCA assays, with inhibition values between 20 and 30%, and an important ability to reduce ferrocyanide, with values around 70 and 80%.

The acetone extracts obtained from *H. hispanica* HM1 presented high antioxidant activity when assayed on ABTS (88%; IC_50_ = 3.89 μg mL^−1^) or DPPH (90%; IC_50_ = 2.06 μg mL^−1^), which was even higher than that of the BHT positive control, however showed medium (56%) or low (30%) antioxidant activity when assayed on FRP or CCA, respectively. Acetone extracts from *H. salinarum* HM2, on the other hand, exhibited a high antioxidant activity when assayed with DPPH (66%; IC_50_ = 8.90 μg mL^-1^) and only low (20–30%) or medium (50%) activity when tested on the other antioxidant assays, showing that differences can be observed between the same type of fractions obtained from both tested haloarchaea.

In addition, the aqueous extracts, obtained from the acetone precipitate of *H. hispanica* HM1, exhibited higher antioxidant activity in FRP assay (82%; EC_50_ = 3.12 μg mL^−1^) than the same extract from *H. salinarum* HM2 (75%; EC_50_ = 3.59 μg/mL). These results suggest that *H. hispanica* HM1 extracts possess a higher antioxidant potential than the *H. salinarum* HM2 ones.

The results obtained entail a complete study about the antioxidant capacity of haloarchaea, as a wide range of antioxidant assays have been employed. In agreement with the results obtained for the new two strains isolated, *H. hispanica* HM1 and *H. salinarum* HM2, extracts from several haloarchaeal species have been reported to show antioxidant activity in the DPPH assay, which is the test most commonly used for this purpose. Examples of this are *Halogeometricum rufum*, *Halogeometricum limi*, *Haladaptatus litoreus*, *Haloplanus vescus*, *Halopelagius inordinatus*, *Halogranum rubrum*, and *Haloferax volcanii,* whose IC_50_ values varied from 2.5 to 10 μg mL^−1^ [12] and *Haloterrigena turkmenica* with an IC_50_ of 4.49 μg mL^−1^ [57]. Regarding the aqueous protein-rich extracts, there are no available reports for comparison in the case of haloarchaea. The fact that different extracts from the studied archaea showed antioxidant activity makes us think that haloarchaea could have antioxidant compounds of different nature and polarity, present in different fractions. Polyphenolic compounds are usually related to the antioxidant activity of plants and algal extracts, [58,59,60]; however there is no evidence to the date of their production in haloarchaea, which antioxidant potential is mainly attributed to the presence of carotenoid pigments. Finally, moderate levels of copper ions chelating activity was found in extracts from both strains. This activity has been previously reported in saline microalgae [61], but has not been studied in haloarchaeal members as yet. Taken together, those results indicate that haloarchaeal extracts could be a promising source of antioxidant compounds with widespread applications as natural food preservatives, colorants and supplements [62], as well as sources of leads for pharmaceutical and cosmetic formulations to prevent oxidative damage [63].

#### 3.2.2. In Vitro Neuroprotective, Antidiabetic, Melanogenic, and Anti-Inflammatory Properties of the Haloarchaeal Extracts

All the extracts were tested to determine their potential activity over the enzymes tyrosinase (TYRO), α-glucosidase, α-amylase, cyclooxygenase 2 (COX-2) and acetylcholinesterase (AChE), which are classical targets in the search for new drug leads for the treatment of diverse dermatological, metabolic, inflammatory, and neurological diseases.

**Figure 3:**
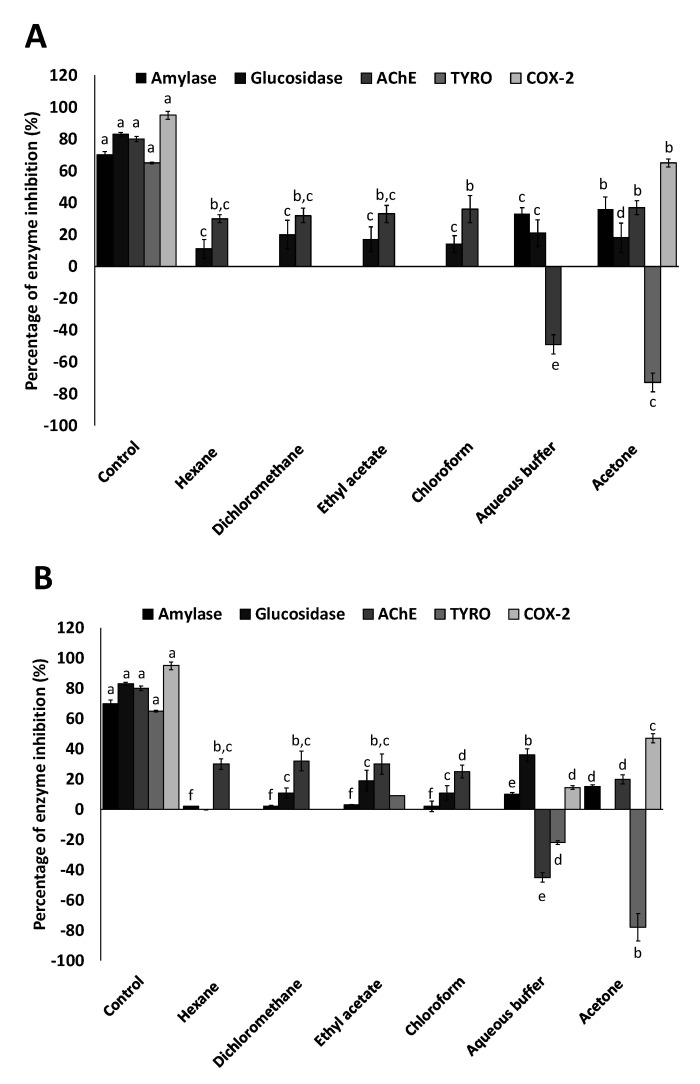
In vitro activity of haloarchaeal extracts on enzymes related to diabetic, neurodegenerative skin pigmentation, and inflammatory diseases. Percentages of inhibition of the enzymes: α-amylase, α-glucosidase, acetylcholinesterase (AChE), tyrosinase (TYRO) and cyclooxygenase 2 (COX-2) by the all extracts obtained in different solvents (hexane, dichloromethane, ethyl acetate, chloroform, aqueous buffer and acetone) from *H. hispanica* HM1 (**A**) and *H. salinarum* HM2 (**B**) strains are represented. A known inhibitor for each enzyme was included as control. For each assay, the data were submitted to one-way variance analysis (ANOVA). Bars are followed by different superscript letters (a–f), which denote groups with significant differences according to the Duncan’s Multiple Range Test (*p* < 0.05).

Among all the tested enzymes, the highest percentage of activity inhibition was observed for COX-2, specifically in the acetone fraction of both haloarchaea, being the *H. hispanica* HM1 extracts more active, with a 65% of enzyme inhibition, than the same extracts of *H. salinarum* HM2, with 47% of COX-2 inhibition. Also, a slight inhibition of COX-2, around 15%, was found in the aqueous fractions of *H. salinarum* HM2 (Figure 3). COX is the central enzyme in the biosynthetic pathway of prostanoids, which are important biological mediators as prostaglandins, prostacyclin, and thromboxane. There are two known isoenzymes: COX-1 and COX-2. COX-1 is constitutively expressed in many tissues and is the predominant form in gastric mucosa and in kidney, while COX-2 is not expressed under normal conditions in most cells, but elevated levels are found during inflammation. Coxibs include a variety of powerful drugs, whose primary mechanism is the potent inhibition of the COX-2 enzyme. These drugs are used worldwide to treat diverse medical conditions as muscular skeletal pain and inflammation. Unfortunately, their use is limited due to negative effects on renal clearance and increased risk of cardiovascular pathology [64]. Therefore, there is a clear need to develop new COX-2 inhibitors free of the aforementioned problems, which can provide pain and inflammatory relief.

Regarding the antidiabetic potential, the aqueous and acetone extracts obtained from *H. hispanica* HM1 biomass exhibited inhibitory activities of 33 and 36%, respectively, on α-amylase activity. In the same way, glucosidase inhibition of 36% was achieved with the aqueous protein-rich extract of *H. salinarum* HM2. Both carbohydrate-hydrolyzing enzymes, α-glucosidase and α-amylase, have been proposed as targets to control the hyperglycemia associated with diabetes mellitus type 2 [65]. The inhibitory potential of these extracts was lower than that of the reference antidiabetic acarbose, which was about 65% for α-amylase and 80% for α-glucosidase (Figure 3).

With respect to the neuroprotective assay, acetone extracts obtained from the biomass of both archaea and all the extracts recovered from culture media showed the capacity of inhibiting acetylcholinesterase activity in a range from 20 to 37%, which was lower than the AChE inhibitory potential of the positive control, galantamine (78%). Conversely, aqueous extracts from both strains exhibited the antagonist activity; they were able to induce the activity of the enzyme by 45%–49% (Figure 3). Acetylcholinesterase catalyzes the hydrolysis of cholinergic neurotransmitters such as acetylcholine and others choline esters to terminate the synaptic transmission rapidly. The reversible inhibition of this enzyme is being explored as treatment for Alzheimer’s disease [66], while acetylcholinesterase activation is required in cholinergic crisis related to acetylcholine accumulations [67].

Regarding tyrosinase activity, none of the extracts studied showed a significant ability to inactivate this oxidase involved in the synthesis of melanin. However, it is remarkable that the acetone extracts from both strains exhibited, in our experimental conditions, a high percentage of tyrosinase activation (73–78%). Although inhibitors of tyrosinase are used to alleviate skin hyperpigmentation and prevent age spots [68], inducers of this enzyme can stimulate melanogenesis and contribute to treat skin depigmentation disorders, such as vitiligo [69,70]. Many melanogenesis stimulators have been isolated from natural sources, but to our knowledge, this is the first report about the influence of archaeal extracts on tyrosinase activity. High repigmentation ratios of vitiligo patients treated with Dead Sea Climatotherapy, which includes sea baths, have been reported [71]. The mineral composition of these saline waters seems to have positive effects in this and other skin conditions; however, the possible therapeutic role of the haloarchaea, which are usual inhabitants of hypersaline water, has not been investigated yet [72]. Furthermore, Vitiligo has also been found to be related to oxidative stress and to the activation/deactivation of acetylcholinesterase [73], and we have demonstrated that haloarchaeal extracts show antioxidant activity and the ability to induce acetylcholinesterase in vitro.

In this study, we have demonstrated that haloarchaeal extracts have interesting bioactive properties. Their ability to inhibit the COX-2 enzyme is especially remarkable, and although in vitro antidiabetic properties of the studied extracts were low, their ability to induce acetylcholinesterase and tyrosinase are noteworthy. To our knowledge, these results represent the first report on the potential applicability of haloarchaeal extracts as a potential source of compounds for the treatment of the aforementioned diseases. To date, the applicability of haloarchaea has been mainly focused on their carotenoids, which have been described as antiproliferative [20] and erythroprotective agents [12], and enhancers of sperm cells viability [13].

### 3.3. Antimicrobial Activity and MIC Determination

The antimicrobial activity of the different extracts obtained from the two new haloarchaeal strains was assayed against a collection of typical fish and human pathogenic bacteria, against representative microalgae and yeast species and over other haloarchaea typically found in hypersaline environments, by using the agar diffusion method.

**Figure 4:**
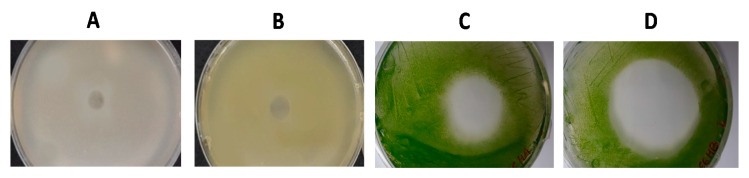
Antimicrobial activity of acetone extracts. Antimicrobial activity was indicated by zones of inhibition of growth or halos. Taking into account the diameter of the halo, the susceptibility of the different microorganisms to the extracts was classified in four reference groups: (**A**) 0.5–1 cm (+) in *Bacillus cereus*; (**B**) 1–2 cm (++) in *Staphylococcus aureus*; (**C**) 2–4 cm (+++) in *Dunaliella bardawil* and (**D**) 4–6 cm (++++) in *Dunaliella salina*.

This screening revealed that only the acetone extracts showed considerable antimicrobial activity. Acetone extracts from the two tested haloarchaeal strains, *H. hispanica* HM1 and *H. salinarum* HM2, showed to be active against bacteria, microalgae, and archaea, but not on yeasts. Zones of inhibition, with diameters which varied according to the susceptibility of the target microorganism from 0.5 to 6 cm, were used as indicators of antimicrobial activity (Figure 4). Both acetone extracts inhibited the growth of all the human pathogenic bacteria assayed and of most fish pathogenic bacteria studied, with inhibition halos ranging from 0.5 to 2 cm. In addition, both extracts exhibited important antimicrobial activity against microalgae, which was especially pronounced over halophilic microalgae from the *Dunaliella* genus, with inhibition halos sized from 2 to 6 cm. Moreover, high activity was found against all the tested extreme halophilic archaea and bacteria, with inhibition zones from 0.5 to 4 cm (Table 1).

**Figure 5:**
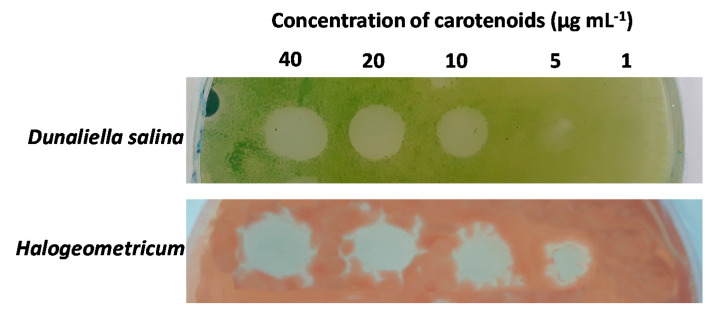
Minimal Inhibitory Concentration plate assay. Dilutions of the active extracts were assayed against one of the most susceptible microorganisms of each group. Halophilic microorganisms, represented by *Dunaliella salina* and *Halogeometricum,* showed to be more sensitive to the extracts.

Several dilutions of acetone extracts containing pigment concentrations of 100, 80, 40, 20, 10, 5 and 1 µg mL^−1^ were prepared to detect their MIC on different target microorganisms. For this purpose, one of the most susceptible microorganisms of each group was selected; *Micrococcus luteus* for human pathogenic bacteria, *Vibrio harveyi* for fish pathogenic bacteria, *Halogeometricum* for archaea, and *Dunaliella salina* for microalgae. The obtained results revealed that the extracts were more active against halophilic microorganisms, with MIC values of 10 µg mL^−1^ for *D. salina* and 5 µg mL^−1^ for *Halogeometricum* (Figure 5); while for the bacteria species *M. luteus* and *V. harveyi*, MIC values were 100 µg mL^−1^.

Lipophilic extracts and pigments of diverse origin have shown antimicrobial activity against pathogenic human bacteria and fungi. In this sense, several studies have been carried out with positive results with pigments derived from plants and vegetables [59,74]; from algae [75,76] and from saline and halophilic bacteria [21,23,77], but to date the reports about the applicability of such extracts as antimicrobial agents are scarce. Recent studies report a moderate inhibitory effect of the extracts from the haloarchaea *Halorubrum* sp. on pathogenic bacteria, with inhibition halos up to 15 mm [24], which is quite lower than the results obtained is this work. Most studies about antimicrobial interactions between halophilic bacteria and archaea are focused on halocins [78,79,80]. Therefore, the present research entails a pioneer report on the effectiveness of haloarchaeal acetonic extracts against a wide range of different microorganisms belonging to the three domains of life. Likewise, this study represents the first outcome from the use of *Haloarcula* and *Halobacterium* as source of antimicrobial compounds.

## 4. Conclusions

This research explores the antioxidant, antimicrobial and other bioactive properties of the extracts obtained from two new isolated haloarchaea strains and confirms that they can be an exceptional source of new bioactive extracts, able to modulate the activity of several medically relevant enzymes, and with potential applicability in pharmacy and cosmetics. The acetone fractions, showed to be the most active extracts in most of the assays, being their antioxidant, antimicrobial, and anti-inflammatory potential especially remarkable. Moreover, acetone extracts from the two haloarchaea have shown for the first time to have the ability to activate the melanogenic enzyme tyrosinase and to inhibit the enzyme cyclooxygenase-2 (COX-2), implicated in the inflammatory response; while the aqueous extracts from both strains have demonstrated to enhance in vitro the acetylcholinesterase activity; this enzyme catalyzes the hydrolysis of cholinergic neurotransmitters and has been reported to be involved in neurological disorders and to have a role in apoptosis. In this study, we have untapped novel unexplored bioactivities of haloarchaea, which can be the source of potential new therapeutic compounds. However, many archaea species are still undiscovered, many potential bioactivities remain to be studied, and the isolation and identification of the compounds responsible for these activities is still a pending issue. Further work will be carried out to fully characterize the composition of the active extracts and their efficacy in in vivo assays.

## Figures and Tables

**Table 1:** Antimicrobial activity of acetone extracts.

	Target Microorganisms	Acetone Extract	
		*H. hispanica* HM1	*H. salinarum* HM2
**Human pathogenic bacteria**	*Bacillus cereus*	**+**	**+**
	*Escherichia coli*	**+**	**−**
	*Micrococcus luteus*	**+**	**++**
	*Staphylococcus aureus*	**+**	**+**
**Marine fish pathogenic bacteria**	*Aeromonas salmonicida* CECT 894T	**−**	**−**
	*Edwardsiella tarda CECT 849T*	**−**	**−**
	*Lactococcus garvieae* CECT 4531T	**−**	**−**
	*Nocardia seriolae* DSM 44129T	**−**	**−**
	*Photobacterium damsela damselae* CECT 626T	**+**	**++**
	*Pseudomonas anguilliseptica CECT 899T*	**++**	**++**
	*Pseudomonas baetica* A390T	**−**	**−**
	*Pseudomonas moraviensis DSM 16007T*	**−**	**−**
	*Pseudomonas plecoglossicida DSM 15088T*	**−**	**−**
	*Streptococcus iniae* CECT 7363T	**−**	**−**
	*Streptococcus parauberis* DSM 6631T	**−**	**+**
	*Tenacibaculum soleae CECT 7292T*	**+**	**−**
	*Vibrio aestuarianus CECT 625T*	**−**	**−**
	*Vibrio anguillarum CECT 522T*	**+**	**+**
	*Vibrio harveyi CECT 525T*	**++**	**++**
	*Vibrio tapetis CECT 4600T*	**+**	**++**
	*Yersinia ruckeri* CECT 4319T	**+**	**+**
**Halophilic archaea and bacteria**	*Haloferax lucentense*	**+**	**++**
	*Haloferax mediterranei*	**+++**	**+++**
	*Halogeometricum*	**+++**	**+++**
	*Halogiper*	**++**	**++**
	*Halorubrum*	**++**	**+++**
	*Haloterrigena*	**++**	**++**
	*Natrinema*	**+**	**+**
	*Salinibacter ruber*	**+++**	**+++**
**Microalgae**	*Chlamydomonas reinhardtii* 21GR	**+**	**++**
	*Chlorella sorokiniana*	**+**	**++**
	*Dunaliella bardawil*	**+++**	**++++**
	*Dunaliella salina*	**+++**	**++++**
**Yeasts**	*Rhodospirillum toruloides* 1854	**−**	**−**
	*Rhodotorula* sp.	**−**	**−**
	*Saccharomyces cerevisiae* F13A	**−**	**−**

Microbial susceptibility was tested by agar diffusion assays and expressed as a function of the diameter of the growth inhibition zone: halo from 0.5 to 1 cm (+), halo from 1 to 2 cm (++), halo from 2 to 4 cm (+++) and halo from 4 to 6 cm (++++); absence of susceptibility (−). For a visual aspect of the halos, please refer to Figure 4.

## Supplementary Materials

The following are available online at https://www.mdpi.com/2079-7737/9/9/298/s1, Figure S1: Sequences of the 16S rRNA genes amplified from the new haloarchaea.

## References

[1] Oren A. (2015). Halophilic microbial communities and their environments. Curr. Opin. Biotechnol..

[2] Ventosa A., Fernández A.B., León M.J., Sánchez-Porro C., Rodriguez-Valera F. (2014). The Santa Pola saltern as a model for studying the microbiota of hypersaline environments. Extremophiles.

[3] Singh A., Singh A.K. (2017). Haloarchaea: Worth exploring for their biotechnological potential. Biotechnol. Lett..

[4] Oren A. (2010). Industrial and environmental applications of halophilic microorganisms. Environ. Technol..

[5] Corral P., Amoozegar M.A., Ventosa A. (2020). Halophiles and their biomolecules: Recent advances and future applications in biomedicine. Mar. Drugs.

[6] Amoozegar M.A., Siroosi M., Atashgahi S., Smidt H., Ventosa A. (2017). Systematics of haloarchaea and biotechnological potential of their hydrolytic enzymes. Microbiol. (UK).

[7] Desai C., Patel P., Markande A.R., Kamala K., Sivaperumal P. (2020). Exploration of haloarchaea for their potential applications in food industry. Int. J. Environ. Sci. Technol..

[8] Besse A., Peduzzi J., Rebuffat S., Carré-Mlouka A. (2015). Antimicrobial peptides and proteins in the face of extremes: Lessons from archaeocins. Biochimie.

[9] Litchfield C.D. (2011). Potential for industrial products from the halophilic Archaea. J. Ind. Microbiol. Biotechnol..

[10] van den Berg H., Faulks R., Granado H.F., Hirschberg J., Olmedilla B., Sandmann G., Southon S., Stahl W. (2000). The potential for the improvement of carotenoid levels in foods and the likely systemic effects. J. Sci. Food Agric..

[11] Bakker M.F., Peeters P.H.M., Klaasen V.M., Bueno-De-Mesquita H.B., Jansen E.H.J.M., Ros M.M., Travier N., Olsen A., Tjønneland A., Overvad K. (2016). Plasma carotenoids, Vitamin C, tocopherols, and retinol and the risk of breast cancer in the European Prospective Investigation into Cancer and Nutrition cohort. Am. J. Clin. Nutr..

[12] Hou J., Cui H.L. (2018). In Vitro Antioxidant, Antihemolytic, and Anticancer Activity of the Carotenoids from Halophilic Archaea. Curr. Microbiol..

[13] Zalazar L., Pagola P., Miró M.V., Churio M.S., Cerletti M., Martínez C., Iniesta-Cuerda M., Soler A.J., Cesari A., De Castro R. (2019). Bacterioruberin extracts from a genetically modified hyperpigmented Haloferax volcanii strain: Antioxidant activity and bioactive properties on sperm cells. J. Appl. Microbiol..

[14] Kirti K., Amita S., Priti S., Mukesh Kumar A., Jyoti S. (2014). Colorful World of Microbes: Carotenoids and Their Applications. Adv. Biol..

[15] Fang C.J., Ku K.L., Lee M.H., Su N.W. (2010). Influence of nutritive factors on C 50 carotenoids production by Haloferax mediterranei atcc 33500 with two-stage cultivation. Bioresour. Technol..

[16] Yatsunami R., Ando A., Yang Y., Takaichi S., Kohno M., Matsumura Y., Ikeda H., Fukui T., Nakasone K., Fujita N. (2014). Identification of carotenoids from the extremely halophilic archaeon Haloarcula japonica. Front. Microbiol..

[17] de la Vega M., Sayago A., Ariza J., Barneto A.G., León R. (2016). Characterization of a bacterioruberin-producing Haloarchaea isolated from the marshlands of the Odiel river in the southwest of Spain. Biotechnol. Prog..

[18] Yang Y., Yatsunami R., Ando A., Miyoko N., Fukui T., Takaichi S., Nakamura S. (2015). Complete biosynthetic pathway of the C50 carotenoid bacterioruberin from lycopene in the extremely halophilic archaeon Haloarcula japonica. J. Bacteriol..

[19] Giani M., Garbayo I., Vílchez C., Martínez-Espinosa R.M. (2019). Haloarchaeal carotenoids: Healthy novel compounds from extreme environments. Mar. Drugs.

[20] Abbes M., Baati H., Guermazi S., Messina C., Santulli A., Gharsallah N., Ammar E. (2013). Biological properties of carotenoids extracted from Halobacterium halobium isolated from a Tunisian solar saltern. BMC Complement. Altern. Med..

[21] Ibrahim D., Nazari T.F., Kassim J., Lim S.H. (2014). Prodigiosin—an antibacterial red pigment produced by Serratia marcescens IBRL USM 84 associated with a marine sponge Xestospongia testudinaria. J. Appl. Pharm. Sci..

[22] Falaise C., François C., Travers M.A., Morga B., Haure J., Tremblay R., Turcotte F., Pasetto P., Gastineau R., Hardivillier Y. (2016). Antimicrobial compounds from eukaryotic microalgae against human pathogens and diseases in aquaculture. Mar. Drugs.

[23] Fariq A., Yasmin A., Jamil M. (2019). Production, characterization and antimicrobial activities of bio-pigments by Aquisalibacillus elongatus MB592, Salinicoccus sesuvii MB597, and Halomonas aquamarina MB598 isolated from Khewra Salt Range, Pakistan. Extremophiles.

[24] Sahli K., Gomri M.A., Esclapez J., Gómez-Villegas P., Ghennai O., Bonete M.J., León R., Kharroub K. (2020). Bioprospecting and characterization of pigmented halophilic archaeal strains from Algerian hypersaline environments with analysis of carotenoids produced by *Halorubrum* sp. BS2. J. Basic Microbiol..

[25] Gómez-Villegas P., Vigara J., León R. (2018). Characterization of the Microbial Population Inhabiting a Solar Saltern Pond of the Odiel Marshlands (SW Spain). Mar. Drugs.

[26] Altschul S.F., Gish W., Miller W., Myers E.W., Lipman D.J. (1990). Basic local alignment search tool. J. Mol. Biol..

[27] Hiyama T., Nishimura M., Chance B. (1969). Determination of carotenes by thin-layer chromatography. Anal. Biochem..

[28] Bradford M.M. (1976). A rapid and sensitive method for the quantitation of microgram quantities of protein utilizing the principle of protein-dye binding. Anal. Biochem..

[29] Sachindra N.M., Sato E., Maeda H., Hosokawa M., Niwano Y., Kohno M., Miyashita K. (2007). Radical scavenging and singlet oxygen quenching activity of marine carotenoid fucoxanthin and its metabolites. J. Agric. Food Chem..

[30] Re R., Pellegrini N., Proteggente A., Pannala A., Yang M., Rice-Evans C. (1999). Antioxidant activity applying an improved ABTS radical cation decolorization assay. Free Radic. Biol. Med..

[31] Baliga M.S., Jagetia G.C., Rao S.K., Babu S.K. (2003). Evaluation of nitric oxide scavenging activity of certain spices in vitro: A preliminary study. Nahr. Food.

[32] Tundis R., Bonesi M., Sicari V., Pellicanò T.M., Tenuta M.C., Leporini M., Menichini F., Loizzo M.R. (2016). Poncirus trifoliata (L.) Raf.: Chemical composition, antioxidant properties and hypoglycaemic activity via the inhibition of α-amylase and α-glucosidase enzymes. J. Funct. Foods.

[33] Megías C., Pastor-Cavada E., Torres-Fuentes C., Girón-Calle J., Alaiz M., Juan R., Pastor J., Vioque J. (2009). Chelating, antioxidant and antiproliferative activity of Vicia sativa polyphenol extracts. Eur. Food Res. Technol..

[34] Iauk L., Acquaviva R., Mastrojeni S., Amodeo A., Pugliese M., Ragusa M., Loizzo M.R., Menichini F., Tundis R. (2015). Antibacterial, antioxidant and hypoglycaemic effects of *Thymus capitatus* (L.) Hoffmanns. et Link leaves’ fractions. J. Enzym. Inhib. Med. Chem..

[35] Orhan I., Kartal M., Naz Q., Ejaz A., Yilmaz G., Kan Y., Konuklugil B., Şener B., Iqbal Choudhary M. (2007). Antioxidant and anticholinesterase evaluation of selected Turkish Salvia species. Food Chem..

[36] Nerya O., Vaya J., Musa R., Izrael S., Ben-Arie R., Tamir S. (2003). Glabrene and isoliquiritigenin as tyrosinase inhibitors from licorice roots. J. Agric. Food Chem..

[37] Johnson M.K., Johnson E.J., MacElroy R.D., Speer H.L., Bruff B.S. (1968). Effects of Salts on the Halophilic Alga Dunaliella viridis1. J. Bacteriol..

[38] Shariati M., Hadi M.R., Capri A. (2011). Microalgal Biotechnology and Bioenergy in Dunaliella. Progress in Molecular and Environmental Bioengineering—From Analysis and Modeling to Technology Applications.

[39] Torreblanca M., Rodriguez-Valera F., Juez G., Ventosa A., Kamekura M., Kates M. (1986). Classification of Non-alkaliphilic Halobacteria Based on Numerical Taxonomy and Polar Lipid Composition, and Description of Haloarcula gen. nov. and Haloferax gen. nov. Syst. Appl. Microbiol..

[40] Oren A., Arahal D.R., Ventosa A. (2009). Emended descriptions of genera of the family Halobacteriaceae. Int. J. Syst. Evol. Microbiol..

[41] Elazari-Volcani B., Breed R.S., Murray EGD S.N. (1957). Genus XII. Halobacterium. Bergey’s Manual of Determinative Bacteriology.

[42] Sneath P.H.A., Mcgowan V., Skerman V.B.D. (1980). Approved Lists of Bacterial Names. Int. J. Syst. Evol. Microbiol..

[43] Yang Y., Cui H.-L., Zhou P.-J., Liu S.-J. (2007). Haloarcula amylolytica sp. nov., an extremely halophilic archaeon isolated from Aibi salt lake in Xin-Jiang, China. Int. J. Syst. Evol. Microbiol..

[44] Ihara K., Watanabe S., Tamura T. (1997). *Haloarcula argentinensis* sp. nov. and *Haloarcula mukohataei* sp. nov., two new extremely halophilic archaea collected in Argentina. Int. J. Syst. Bacteriol..

[45] Gonzalez C., Gutierrez C., Ramirez C. (1978). Halobacterium vallismortis sp. nov. An amylolytic and carbohydrate-metabolizing, extremely halophilic bacterium. Can. J. Microbiol..

[46] Oren A., Ginzburg M., Ginzburg B.Z., Hochstein L.I., Volcani B.E. (1990). Haloarcula marismortui (Volcani) sp. nov., nom. rev., an extremely halophilic bacterium from the Dead Sea. Int. J. Syst. Bacteriol..

[47] Juez G., Rodriguez-Valera F., Ventosa A., Kushner D.J. (1986). *Haloarcula hispanica* spec. nov. and *Haloferax gibbonsii* spec, nov., two new species of extremely halophilic archaebacteria. Syst. Appl. Microbiol..

[48] Takashina T., Hamamoto T., Otozai K., Grant W.D., Horikoshi K. (1990). *Haloarcula japonica* sp. nov., a New Triangular Halophilic Archaebacterium. Syst. Appl. Microbiol..

[49] Oren A., Ventosa A., Gutiérrez M.C., Kamekura M. (1999). *Haloarcula quadrata* sp. nov., a square, motile archaeon isolated from a brine pool in Sinai (Egypt). Int. J. Syst. Evol. Microbiol..

[50] Javor B., Requadt C., Stoeckenius W. (1982). Box-shaped halophilic bacteria. J. Bacteriol..

[51] Namwong S., Tanasupawat S., Kudo T., Itoh T. (2010). *Haloarcula salaria* sp. nov. and *Haloarcula tradensis* sp. nov. from salt in Thai fish sauce. Int. J. Syst. Evol. Microbiol..

[52] Yang Y., Cui H.-L., Zhou P.-J., Liu S.-J. (2006). *Halobacterium jilantaiense* sp. nov., a halophilic archaeon isolated from a saline lake in Inner Mongolia, China. Int. J. Syst. Evol. Microbiol..

[53] Lü Z.-Z., Li Y., Zhou Y., Cui H.-L., Li Z.-R. (2017). *Halobacterium litoreum* sp. nov., isolated from a marine solar saltern. Int. J. Syst. Evol. Microbiol..

[54] Gruber C., Legat A., Pfaffenhuemer M., Radax C., Weidler G., Busse H.-J., Stan-Lotter H. (2004). *Halobacterium noricense* sp. nov., an archaeal isolate from a bore core of an alpine Permian salt deposit, classification of *Halobacterium* sp. NRC-1 as a strain of *H. salinarum* and emended description of *H. salinarum*. Extremophiles.

[55] Han D., Cui H.-L. (2014). *Halobacterium rubrum* sp. nov., isolated from a marine solar saltern. Arch. Microbiol..

[56] Ventosa A., Oren A. (1996). *Halobacterium salinarum* nom. corrig., a name to replace *Halobacterium salinarium* (Elazari-Volcani) and to include *Halobacterium halobium* and *Halobacterium cutirubrum*. Int. J. Syst. Evol. Microbiol..

[57] Squillaci G., Parrella R., Carbone V., Minasi P., La Cara F., Morana A. (2017). Carotenoids from the extreme halophilic archaeon Haloterrigena turkmenica: Identification and antioxidant activity. Extremophiles.

[58] Huynh Thi Le D., Lu W.-C., Li P.-H. (2020). Sustainable Processes and Chemical Characterization of Natural Food Additives: Palmyra Palm (Borassus Flabellifer Linn.) Granulated Sugar. Sustainability.

[59] Szabo K., Diaconeasa Z., Cătoi A.F., Vodnar D.C. (2019). Screening of ten tomato varieties processing waste for bioactive components and their related antioxidant and antimicrobial activities. Antioxidants.

[60] Maadane A., Merghoub N., Ainane T., El Arroussi H., Benhima R., Amzazi S., Bakri Y., Wahby I. (2015). Antioxidant activity of some Moroccan marine microalgae: Pufa profiles, carotenoids and phenolic content. J. Biotechnol..

[61] Custódio L., Silvestre L., Rocha M.I., Rodrigues M.J., Vizetto-Duarte C., Pereira H., Barreira L., Varela J. (2016). Methanol extracts from *Cystoseira tamariscifolia* and *Cystoseira nodicaulis* are able to inhibit cholinesterases and protect a human dopaminergic cell line from hydrogen peroxide-induced cytotoxicity. Pharm. Biol..

[62] Chaari M., Theochari I., Papadimitriou V., Xenakis A., Ammar E. (2018). Encapsulation of carotenoids extracted from halophilic Archaea in oil-in-water (O/W) micro- and nano-emulsions. Colloids Surf. B Biointerfaces.

[63] Jaswir I., Noviendri D., Hasrini R.F., Octavianti F. (2011). Carotenoids: Sources, medicinal properties and their application in food and nutraceutical industry. J. Med. Plant. Res..

[64] D Tortorella M., Zhang Y., Talley J. (2016). Desirable Properties for 3rd Generation Cyclooxygenase-2 Inhibitors. Mini Rev. Med. Chem..

[65] Saeedi M., Hadjiakhondi A., Nabavi S.M., Manayi A. (2017). Heterocyclic Compounds: Effective alpha-Amylase and alpha-Glucosidase Inhibitors. Curr. Top. Med. Chem..

[66] Saxena M., Dubey R. (2019). Target Enzyme in Alzheimer’s Disease: Acetylcholinesterase Inhibitors. Curr. Top. Med. Chem..

[67] Rovenský J., Payer J. (2009). Cholinergic crisis. Dictionary of Rheumatology.

[68] Pillaiyar T., Manickam M., Namasivayam V. (2017). Skin whitening agents: Medicinal chemistry perspective of tyrosinase inhibitors. J. Enzym. Inhib. Med. Chem..

[69] Bae-Harboe Y.-S.C., Park H.-Y. (2012). Tyrosinase: A Central Regulatory Protein for Cutaneous Pigmentation. J. Investig. Dermatol..

[70] Niu C., Aisa H.A. (2017). Upregulation of Melanogenesis and Tyrosinase Activity: Potential Agents for Vitiligo. Molecules.

[71] Czarnowicki T., Harari M., Ruzicka T., Ingber A. (2011). Dead Sea climatotherapy for vitiligo: A retrospective study of 436 patients. J. Eur. Acad. Dermatol. Venereol..

[72] Carbajo J.M., Maraver F. (2018). Salt water and skin interactions: New lines of evidence. Int. J. Biometeorol..

[73] Schallreuter K.U., Elwary S.M.A., Gibbons N.C.J., Rokos H., Wood J.M. (2004). Activation/deactivation of acetylcholinesterase by H_2_O_2_: More evidence for oxidative stress in vitiligo. Biochem. Biophys. Res. Commun..

[74] Boo H.O., Hwang S.J., Bae C.S., Park S.H., Heo B.G., Gorinstein S. (2012). Extraction and characterization of some natural plant pigments. Ind. Crops Prod..

[75] Manilal A., Sujith S., Selvin J., Kiran G.S., Shakir C., Lipton A.P. (2010). Potencial de los antimicrobianos de los organismos marinos de la costa sureste de la India frente a patógenos multirresistentes del camarón y humanos. Sci. Mar..

[76] Karpiński T.M., Adamczak A. (2019). Fucoxanthin—An antibacterial carotenoid. Antioxidants.

[77] Chen L., Wang G., Bu T., Zhang Y., Wang Y., Liu M., Lin X. (2010). Phylogenetic analysis and screening of antimicrobial and cytotoxic activities of moderately halophilic bacteria isolated from the Weihai Solar Saltern (China). World J. Microbiol. Biotechnol..

[78] Atanasova N.S., Pietilä M.K., Oksanen H.M. (2013). Diverse antimicrobial interactions of halophilic archaea and bacteria extend over geographical distances and cross the domain barrier. Microbiologyopen.

[79] Mazguene S., Rossi M., Gogliettino M., Palmieri G., Cocca E., Mirino S., Imadalou-Idres N., Benallaoua S. (2018). Isolation and characterization from solar salterns of North Algeria of a haloarchaeon producing a new halocin. Extremophiles.

[80] Quadri I., Hassani I.I., l’Haridon S., Chalopin M., Hacène H., Jebbar M. (2016). Characterization and antimicrobial potential of extremely halophilic archaea isolated from hypersaline environments of the Algerian Sahara. Microbiol. Res..

