# Supplementary material for: Antioxidant, Antimicrobial, and Bioactive Potential of Two New Haloarchaeal Strains Isolated from Odiel Salterns (Southwest Spain)
Source: Biology (Basel). 2020 Sep 18;9(9):298. doi: 10.3390/biology9090298 (PMC7564620; doi:10.3390/biology9090298)
Supplement: Supplementary file 1 [file biology-09-00298-s001.pdf]

## *Haloarcula hispanica* **HM1, 16S RNA coding gene sequence**

GGAATCGATTAGCCCTGCTAGTCGCACGGGTCTTAGACTCCGTAGGCATATAGCTCAGTAACACGTGGC
CAAACTACCCTACAGACCGCGATAACCTCGGGAAACTGAGGCCAATAGCGGATATAACTCTCAGGCTG
GAGTGCCGAGAGTTAGAAACGTTCCGGCGCTGTAGGATGTGGCTGCGGCCGATTAGGTAGATGGTGG
GGTAACGGCCCACCATGCCGATAATCGGTACGGGTTGTTGGAGAGCAAGAACCCGGAGACGGTATTT
GAGACAAGATACCGGGCCCTACGGGGCGCAGCAGGCGGGAAACCTTTACACTGCACGACAGTGCGAT
AGGGGGACTCCGAGTGTGAGGGCATATAGCCCTCGCTTTTCTGTACCGTAAGGTGGTACAGGAACAAG
GACTGGGCAAGACCGGTGCCAGCCGCCGCGGTAATACCGGCAGTCCAAGTGATGGCCGATATTATTGG
GCCTAAAGCGTCCGTAGCCGGCCGGACAAGTCCGTTGGGAAATCGACGAGCTCAACGCGTCGGCGTCC
AGCGGAAACTGTCCGGCTTGGGGCCGGAAGACTTGGGGGGTACGTCCGGGGTAGGAGTGAAATCCTG
TAATCCTGGACGGACCACCAATGGGGAAACCACCTCAGGAAGCCGGACCCGACGGTGAGGGACGAAA
GCCAGGGTCTCGAACCGGATTAGATACCCGGGTAGTCCTAGCTGTAAACGATGCTCGCTAGGTGTGCC
GTAGGCCACGAGCATGCGATGCGCCGTAGGGAAGCCGAGAAGCGAGCCGCCTGGGAAGTACGTCTGC
AAGGATGAAACTTAAAGGAATTGGCGGGGGAGCACCACAACCGGAGGAGCCTGCGGTTTAATTGGAC
TCAACGCCGGAAATCTCACCGGTCCCGACAGTAGTAATGACGGTCAGGTTGACGACTTTACCCGACGCT
ACTGAGAGGAGGTGCATGGCCGCCGTCAGCTCGTACCGTGAGGCGTCCTGTTAAGTCAGGCAACGAG
CGAGACCCGCACTTCTAGTTGCCAGCAATACCCTTGAGGTAGTTGGGTACACTAGGAGGACTGCCGCT
GCTAAAGCGGAGGAAGGAACGGGCAACGGTAGGTCAGTATGCCCCGAATGGACCGGGCAACACGCG
GGCTACAATGGCTCTGACAGTGGGATGCAACGCCGAGAGGCGACGCTAATCTCCAAACGGAGTCGTA
GTTCGGATTGCGGGCTGAAACCCGCCCGCATGAAGCTGGATTCGGTAGTAATCGCGTGTCAGAAGCGC
GCGGTGAATACGTCCCTGCTCCTTGCACACACCGCCCGTCAAAGCACCCGAGTGGGGTCCGGATGAGG
CCGTCATGCGACGGTCGAATCCT

## *Halobacterium salinarum* **HM2, 16S RNA coding gene sequence**

GGATCGATTAGCCATGCTAGTTGTGCGGGTTTAGACCCGCAGCGGAAGCTCAGTAACACGTGGCCAAG
CTACCCTGTGGACGGGAATACTCTCGGGAAACTGAGGCTAATCCCCGATAACGCTTTGCTCCTGGAAG
GGGCAAAGCCGGAAACGCTCCGGCGCCACAGGATGCGGCTGCGGTCGATTAGGTAGACGGTGGGGT
AACGGCCCACCGTGCCCATAATCGGTACGGGTTGTGAGAGCAAGAGCCCGGAGACGGAATCTGAGAC
AAGATTCCGGGCCCTACGGGGCGCAGCAGGCGCGAAACCTTTACACTGTACGAAAGTGCGATAAGGG
GACTCCGAGTGTGAAGGCATAGAGCCTTCACTTTTGTACACCGTAAGGTGGTGCACGAATAAGGACTG
GGCAAGACCGGTGCCAGCCGCCGCGGTAATACCGGCAGTCCGAGTGATGGCCGATCTTATTGGGCCTA
AAGCGTCCGTAGCTGGCTGAACAAGTCCGTTGGGAAATCTGTCCGCTTAACGGGCAGGCGTCCAGCGG
AAACTGTTCAGCTTGGGACCGGAAGACCTGAGGGGTACGTCTGGGGTAGGAGTGAAATCCTGTAATCC
TGGACGGACCGCCGGTGGCGAAAGCGCCTCAGGAGAACGGATCCGACAGTGAGGGACGAAAGCTAG
GGTCTCGAACCGGATTAGATACCCGGGTAGTCCTAGCTGTAAACGATGTCCGCTAGGTGTGGCGCAGG
CTACGAGCCTGCGCTGTGCCGTAGGGAAGCCGAGAAGCGGACCGCCTGGGAAGTACGTCTGCAAGGA
TGAAACTTAAAGGAATTGGCGGGGGAGCACTACAACCGGAGGAGCCTGCGGTTTAATTGGACTCAAC
GCCGGACATCTCACCAGCCCCGACAGTAGTAATGACGGTCAGGTTGATGACCTTACCCGAGGCTACTG
AGAGGAGGTGCATGGCCGCCGTCAGCTCGTACCGTGAGGCGTCCTGTTAAGTCAGGCAACGAGCGAG
ACCCGCACTCCTAATTGCCAGCAGTACCCTTTGGGTAGCTGGGTACATTAGGTGGACTGCCGCTGCCAA
AGCGGAGGAAGGAACGGGCAAGCCCCGAATGGGCTGGGCAACACGCGGGCTACAATGGTCGAGACA
ATGGGAAGCCACTCCGAGAGGAGGCGCTAATCTCCTAAACTCGATCGTAGTTCGGATTGAGGGCTGAA
ACTCGCCCTCATGAAGCTGGATCGGTAGGTCAGTATTCGGTAGTAATCGCGTGTCAGCAGCGCGCGGT
GAATACGTCCCTGCTCCTTGCACACACCGCCCGTCAAATCACCCGAGTGGGGTTCGGATGAGGCCGGC
ATGCGCTGGTCAAATCGGC
